# Exploring the Relationship Between Gut Microbiota and Aortic Stenosis: Role of Inflammatory Proteins, Blood Metabolites, and Immune Cells

**DOI:** 10.7150/ijms.110392

**Published:** 2025-03-10

**Authors:** Fanhui Jing, Jiapeng Zhou, Fengwen Zhang, Guangzhi Zhao, Fang Fang, Xiangbin Pan

**Affiliations:** 1 Department of Structural Heart Disease, National Center for Cardiovascular Disease, Fuwai Hospital, Chinese Academy of Medical Sciences and Peking Union Medical College, Beijing, China.; 2 National Health Commission Key Laboratory of Cardiovascular Regeneration Medicine, Fuwai Hospital, Chinese Academy of Medical Sciences and Peking Union Medical College, Beijing, China.; 3 State Key Laboratory of Cardiovascular Disease, Fuwai Hospital, Chinese Academy of Medical Sciences and Peking Union Medical College, Beijing, China.; 4 Key Laboratory of Innovative Cardiovascular Devices, Chinese Academy of Medical Sciences and Peking Union Medical College, Beijing, China.; 5 National Clinical Research Center for Cardiovascular Diseases, Fuwai Hospital, Chinese Academy of Medical Sciences and Peking Union Medical College, Beijing, China.; 6 College of Life Sciences, Hunan Normal University, Changsha, China.

**Keywords:** Aortic stenosis, Gut microbiota, Inflammatory protein, Blood metabolite, Immune cell, Mendelian randomization analysis

## Abstract

**Background:** Aortic stenosis is the most prevalent valvular heart disease in high-income population, and there are currently no medical therapies to slow the disease progression. Given that gut microbiota influences the immune system, lipid metabolism, and inflammation, there may be a potential link between gut microbiota and AS.

**Aims:** We aimed to examine the causal effects of gut microbiota on AS and to investigate the mediating roles of inflammatory proteins, blood metabolites, and immune cells.

**Methods:** Bidirectional Mendelian randomization analysis was performed to assess the causal relationships between gut microbiota, inflammatory proteins, blood metabolites, immune cells, and AS. Two-step Mendelian randomization was utilized to explore direct and indirect effects. The data were derived from genome-wide association study summary statistics available in public databases.

**Results:** The study identified nine gut microbial features (six microbial taxa and three pathways), four inflammatory proteins, 91 blood metabolites, and four immune cell traits associated with AS. However, no significant mediating roles were found for inflammatory proteins, blood metabolites, and immune cells in the causal pathway between gut microbiota and AS.

**Conclusion:** This study revealed novel causal associations between gut microbial features, inflammatory proteins, blood metabolites, and immune cell traits with AS. These findings offer new insights into the pathophysiology of AS and provide potential targets for therapeutic approaches.

## Introduction

Aortic stenosis (AS) is the most prevalent valvular heart disease in Europe and North America 1-3. Although surgical aortic valve replacement and transcatheter aortic valve implantation are effective for severe AS 1,4, no medical therapies currently exist to slow disease progression. Investigations have identified several modifiable risk factors for AS, such as hypertension 5, obesity 6,7, diabetes 8, dyslipidaemia 9-11, which may give a few hints for future pharmaceutical development. Immune and inflammatory mechanisms were also found to play important roles in the progress of AS 12. Meanwhile, the critical role of gut microbiota in modulating immune system 13, lipid metabolism 14, and inflammation 15 has been verified by many studies. Several studies have explored the connection between gut microbiota and AS 16. Elevated acylcarnitine, choline and TMAO levels, which were closely linked to gut microbial metabolism, have been reported in AS patients 17,18. Liu et al. 19 found distinct gut microbial profiles in AS compared to coronary artery disease. Nevertheless, no study explored the potential causal relationship between gut microbiota and AS at the genetic level.

Mendelian randomization (MR) is a potent epidemiological and genetic research method for exploring the causal links between risk factors and disease outcomes. It relies on Mendelian genetics principles, which dictate that alleles are dispersed at random during gamete formation, mirroring the randomization process in randomized controlled trials. This helps address the issues of reverse causality and confounding commonly seen in observational research 20,21.

In this study, we performed a comprehensive MR analysis to investigate the potential causal effects of gut microbiota on AS, inflammatory proteins on AS, blood metabolites on AS, immune cells on AS, respectively. Then we explored whether inflammatory proteins, blood metabolites, or immune cells serve as mediators in the relationship between gut microbiota and AS.

## Materials and Methods

### Study design

**Figure 1:**
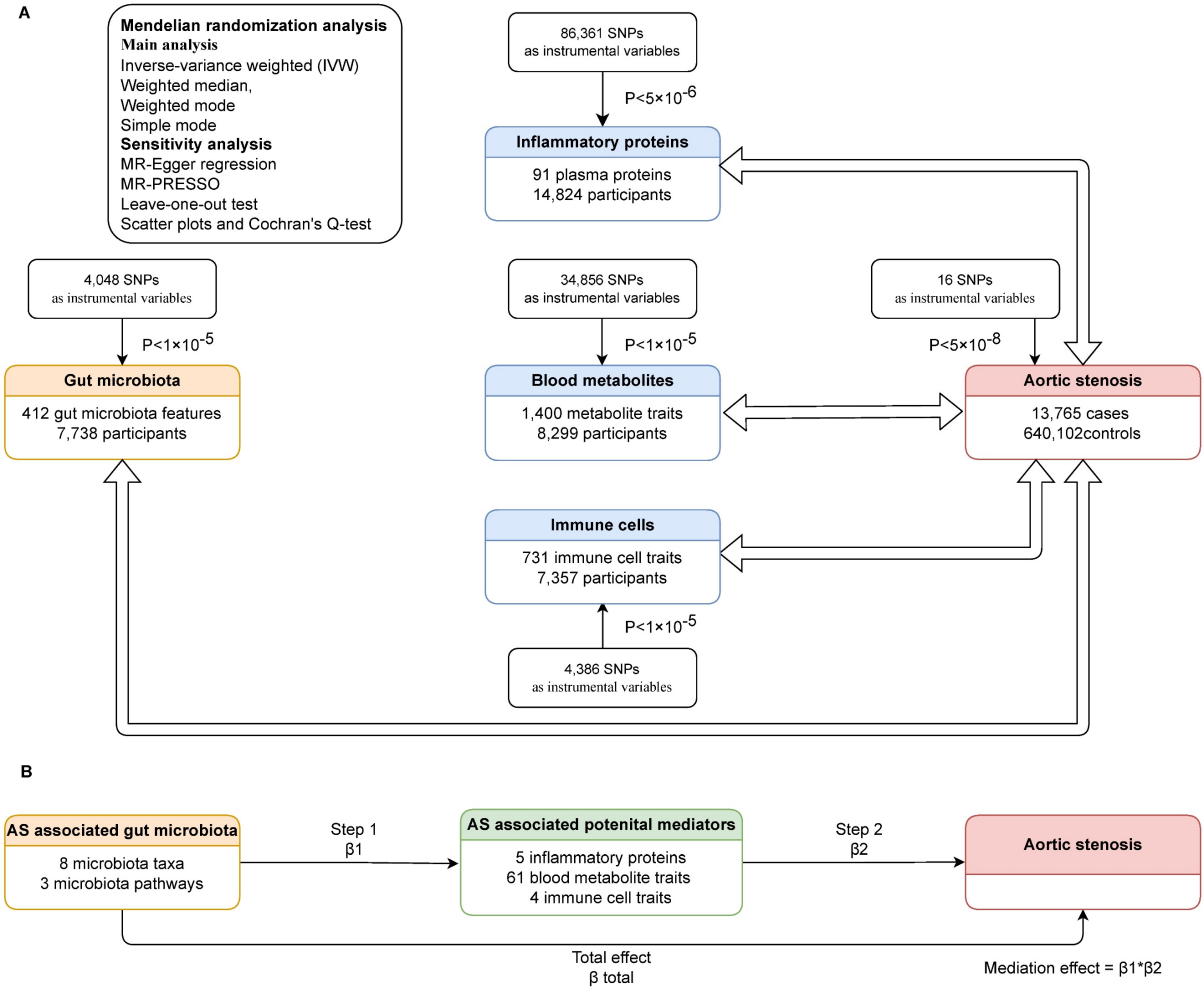
The study design. (A)Bidirectional mendelian randomization between gut microbiota and AS, inflammatory proteins and AS, blood metabolites and AS, immune cells and AS. (B)TSMR to decompose the direct and indirect effects of the gut microbiota and potential mediators on AS. AS: aortic stenosis. TSMR: two-step Mendelian randomization. MR: mendelian randomization. SNP: single nucleotide polymorphism. β1, β2, β total: the causal effect of exposure on outcome in each step.

We carried out bidirectional Mendelian randomization to explore the links between gut microbiota and AS, as well as between inflammatory proteins and AS, blood metabolites and AS, immune cells and AS. Then we assessed whether gut microbiota had a causal impact on potential mediators (inflammatory proteins, blood metabolites and immune cells associated with AS), and if so, we adopted two-step Mendelian randomization (TSMR) to determine the direct and indirect effects of the gut microbiota and potential mediators on AS. In TSMR analysis, we included gut microbiota and potential mediators that have been demonstrated significant associated with AS, using only genetic instrumental variables (IVs) not used in step one, to evaluate the effect of the potential mediator on AS. The mediation effect was calculated only when the direction of the total effect (beta of the gut microbiota-AS association) aligned with the direction of the effect through the mediator (beta of the gut microbiota-potential mediator association × beta of the potential mediator-AS association). The study design is illustrated in the flowchart (Figure 1).

### Data sources

All genome-wide association study (GWAS) summary statistics utilized in this study were sourced from public databases and were accessible, as listed in Table S1. The original studies have already obtained ethics and institutional review board approvals and informed consent was acquired from the participants or caregivers.

Data for AS were sourced from a GWAS meta-analysis of 10 cohorts, involving 13,765 cases and 640,102 controls of European ancestry and revealing 11,591,806 variants 22. Gut microbiota data were derived from a GWAS study conducted by Esteban et al. 23, who analyzed metagenomic sequencing data of 207 microbial taxa and 205 pathways representing microbial composition and function in 7,738 people from the northern Netherlands. The data of inflammation-related proteins were obtained from the study performed by Zhao et al. 24, who carried out a genome-wide protein quantitative trait locus study of 91 plasma proteins in 14,824 participants. Blood metabolites data came from a GWAS study by Yiheng Chen et al. 25, encompassing 1,091 metabolites and 309 metabolite ratios in 8,299 individuals from the Canadian Longitudinal Study on Aging (CLSA) cohort, with 850 metabolites categorized into eight superpathways 25. Immune cell traits data were extracted from the GWAS Catalog (accession numbers from GCST0001391 to GCST0002121). The original GWAS study 26 involved 3,757 European individuals and investigated the impact of approximately 22 million SNPs on 731 immunophenotypes.

### Instrumental variable selection

Candidate IVs for gut microbiota, immune cell traits and blood metabolites were selected based on the genome-wide significance threshold *P* < 1×10^-5^, consistent with previous studies 26-28. To increase the number of IVs for inflammatory proteins, a threshold of *P* < 5×10⁻⁶ was applied. IVs related to AS were chosen at the conventional GWAS threshold of *P* < 5×10⁻⁸.Then we clumped all those IVs using conventional thresholds of 10 Mb and r^2^<0.001 to identify independent IVs free from linkage disequilibrium, utilizing the 1,000 Genomes European reference panel. F statistics were calculated to assess the strength of each SNP as an IV. Weak instruments (F statistic <10) were excluded from analyses to reduce weak instrument bias 29.

### Statistical analysis

The inverse-variance weighted (IVW) method was primarily used in the analysis to evaluate causal relationships between exposures and outcomes 30. Weighted median, weighted mode, and simple mode were applied to test the robustness of the findings. Results were presented as beta (β) value with standard error for the continuous outcomes and odds ratio (OR) with a 95% confidence interval (CI) for the binary outcomes. To address multiple testing, false discovery rate (FDR) correction of *P*-values was applied using the Benjamini-Hochberg method for all IVW results to control Type I errors. Significant results were those with the FDR-adjusted *P*-values < 0.05.

In the sensitivity analysis, pleiotropy and heterogeneity were examined. MR-Egger regression method was utilized as the main estimation to account for potential pleiotropy 31. MR-PRESSO (MR Pleiotropy RESidual Sum and Outlier) was employed to identify and correct for horizontal pleiotropy by removing outliers 32. Leave-one-out test was used to estimate the potential pleiotropic effects of single SNPs. Scatter plots and Cochran's Q-test were used to estimate heterogeneity, and *P* < 0.05 indicates the presence of heterogeneity.

For mediation estimation, we adopted TSMR to identify the direct and indirect effects of the gut microbiota and candidate mediators on AS. The proportion mediated by a candidate mediator was calculated as the estimated effect of the gut microbiota on the candidate mediator multiplied by the estimated effect of the candidate mediator on AS.

All analyses were conducted using the R platform (version 4.2.1). The statistical analyses and data visualizations were performed with the 'TwoSampleMR', 'Mendelian Randomization', 'MRPRESSO' packages.

## Results

### Instrument variables included in analysis

A total of 4,048, 86,361, 34,856, and 4,386 SNPs were selected as IVs for gut microbiota, inflammatory proteins, blood metabolites, and immune cells, respectively. When AS was used as exposure factors, 16 SNPs were selected according to the selection criteria mentioned above. Detailed information of these IVs was shown in Table S2-S6.

### The causal relationship between gut microbiota and AS

**Figure 2:**
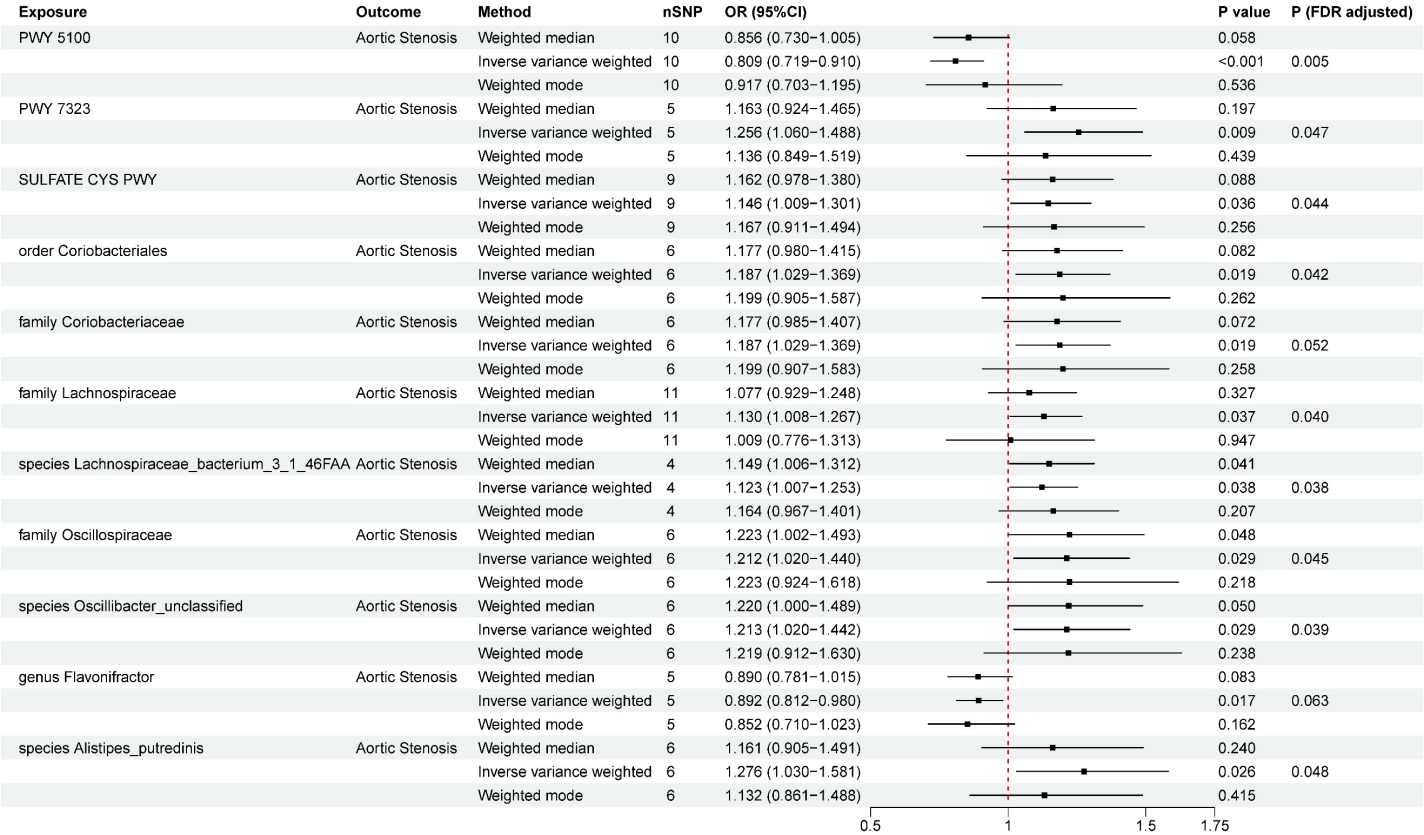
Two-sample Mendelian randomization estimations showing the effect of gut microbiota on aortic stenosis. Only the results of the IVW, weighted median, and weighted mode analysis methods were shown in the figure, and the results of other methods can be found in Table S7. nSNP: total number of instrumental variables used for analysis. PWY 5100: pyruvate fermentation to acetate and lactate II. PWY 7323: superpathway of GDP mannose derived O antigen building blocks biosynthesis. SULFATE CYS PWY: superpathway of sulfate assimilation and cysteine biosynthesis.* P* (FDR adjusted) < 0.05 was considered statistically significant.

A total of eight microbial taxa (including three families, one genus, one order, and three species) and three pathways were identified associated with AS at the nominal significance level of 0.05 (Figure 2). After FDR correction, six microbial taxa (including two families, one order, and three species) and three pathways still remained significant. PWY 5100 (pyruvate fermentation to acetate and lactate II) (OR = 0.809, 95%CI = 0.719~0.910 , *P*_FDR_= 0.005) was associated with an decreased risk of AS, while order *Coriobacteriales* (OR=1.187, 95%CI =1.029~1.369, *P*_FDR_= 0.042), family Coriobacteriaceae (OR=1.187, 95%CI =1.029~1.369, *P*_FDR_= 0.052), family *Lachnospiraceae* (OR = 1.130, 95%CI = 1.008~1.267 , *P*_FDR_= 0.040), species *Lachnospiraceae bacterium 3_1_46FAA* (OR=1.123,95%CI=1.007~1.253, *P*_FDR_= 0.038), family *Oscillospiraceae* (OR = 1.212, 95%CI = 1.020~1.440, *P*_FDR_= 0.045), species* Oscillibacter_unclassified* (OR=1.213,95%CI =1.020~1.442, *P*_FDR_= 0.039)*,* species *Alistipes_putredinis* (OR=1.276,95%CI =1.030~1.581, *P*_FDR_ = 0.048), PWY 7323 (superpathway of GDP-mannose-derived O-antigen building blocks biosynthesis) (OR = 1.256, 95%CI = 1.060~1.488, *P*_FDR_= 0.047), and SULFATE CYS PWY (superpathway of sulfate assimilation and cysteine biosynthesis) (OR = 1.146, 95%CI = 1.009~1.301, *P*_FDR_= 0.044) were at risk of increasing AS. The associations remained consistent in sensitivity analyses, with no heterogeneity or pleiotropy was observed in the primary analysis (Table S7). To test reverse causality, we conducted a reverse MR analysis and found no evidence of associations of genetic liability to AS with identified gut microbiota was identified (Table S8). The results of “leave-one-out” analysis confirmed the reliability of the MR analysis (Figure S1). The scatter plots illustrated the overall effect of gut microbiota on AS (Figure S2).

### The causal relationship between inflammatory proteins and AS

**Figure 3:**
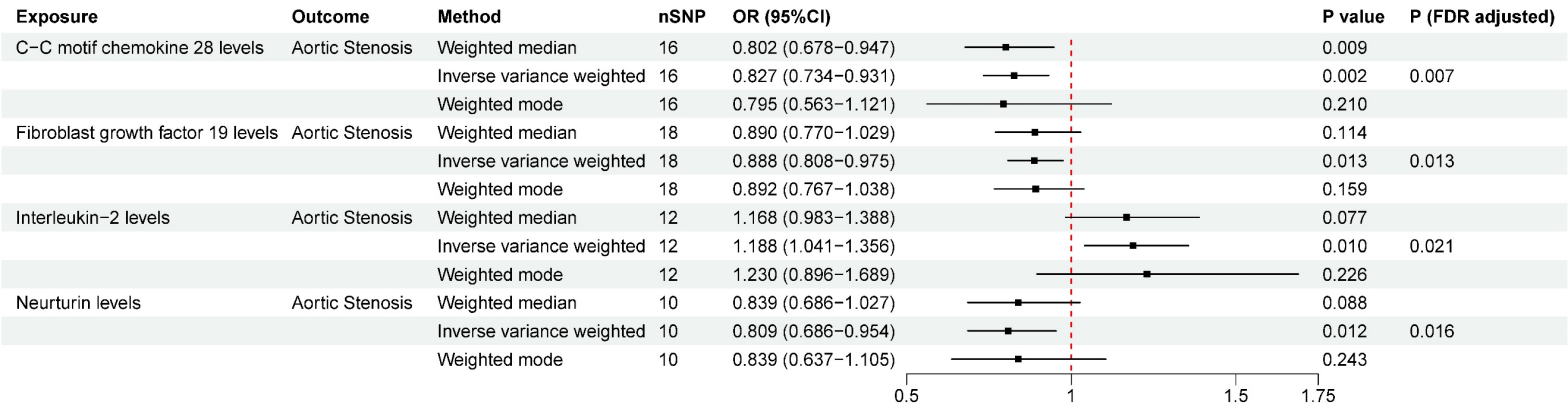
Two-sample Mendelian randomization estimations showing the effect of inflammatory proteins on aortic stenosis. Only the results of the IVW, weighted median, and weighted mode analysis methods were shown in the figure, and the results of other methods can be found in Table S9. nSNP: total number of instrumental variables used for analysis. *P* (FDR adjusted) < 0.05 was considered statistically significant.

We detected significant associations of four inflammatory proteins with AS both at the nominal significance level of 0.05 and after FDR correction. (Figure. 3). C-C motif chemokine ligand 28 (CCL-28) (OR = 0.827, 95%CI = 0.734~0.931, *P*_FDR_= 0.007), fibroblast growth factor 19 (FGF-19) (OR = 0.888, 95%CI = 0.808~0.975, *P*_FDR_= 0.013), and neurtuin (OR = 0.809, 95%CI = 0.686~0.954, *P*_FDR_= 0.016) were associated with a lower risk of AS. While interleukin-2 (IL-2) (OR = 1.188, 95%CI = 1.041~1.356, *P*_FDR_= 0.021) was observed to increase the risk of AS. No significant heterogeneity or horizontal pleiotropy was detected based on Cochrane's Q, MR-Egger, and MR-PRESSO tests. In the reverse MR analysis, we found no evidence linking genetic liability to AS with the levels of identified inflammatory proteins (Table S10). The results of “leave-one-out” analysis proved that MR analysis confirmed the reliability of the MR analysis (Figure S3). The scatter plots demonstrated the overall effect of inflammatory proteins on AS (Figure S4).

### The causal relationship between blood metabolites and AS

**Figure 4:**
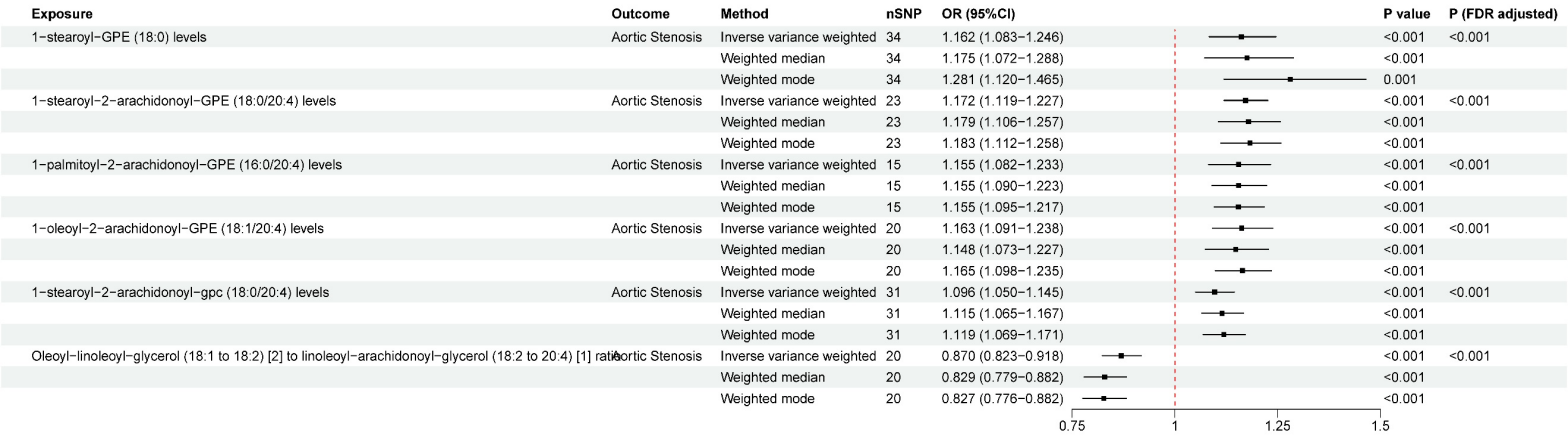
Two-sample Mendelian randomization estimations showing the effect of blood metabolites on aortic stenosis. Only the results of the IVW, weighted median, and weighted mode analysis methods were shown in the figure, and the results of other methods can be found in Table S11. nSNP: total number of instrumental variables used for analysis.* P* (FDR adjusted) < 0.05 was considered statistically significant.

A total of 91 blood metabolites has significant causal associations with AS (Table S11), both at the nominal significance level of 0.05 and after FDR correction. Heterogeneity was observed in 25 metabolites by Cochran's Q-test and 16 metabolites were detected to have horizontal pleiotropy by MR-Egger, and these metabolites were excluded in following analysis. Additionally, six blood metabolites were excluded in the subsequent analysis due to having reverse causal relationship with AS (Table S12). The most significant causal effects were observed between seven blood metabolic traits and AS (Figure. 4). 1-stearoyl-GPE (18:0) (OR =1.162, 95%CI = 1.083~1.246, *P*_FDR_= 5.03×10^-4^), 1-stearoyl-2-arachidonoyl-GPE (18:0/20:4) (OR = 1.171, 95%CI = 1.119~1.227, *P*_FDR_= 1.13×10^-9^), 1-palmitoyl-2-arachidonoyl-GPE (16:0/20:4) (OR = 1.155, 95%CI = 1.082~1.233, *P*_FDR_= 3.57×10^-4^), 1-oleoyl-2-arachidonoyl-GPE (18:1/20:4) (OR = 1.163, 95%CI = 1.091~1.238, *P*_FDR_= 8.88×10^-5^), 1-stearoyl-2-arachidonoyl-gpc (18:0/20:4) (OR = 1.096, 95%CI = 1.050~1.145, *P*_FDR_= 4.21×10^-4^), and Linoleoyl-arachidonoyl-glycerol (18:2/20:4) 2 (OR = 1.150, 95%CI = 1.074-1.232, *P*_FDR_= 8.53×10^-4^) were associated with an increased risk of AS. Oleoyl-linoleoyl-glycerol (18:1 to 18:2) 2 to linoleoyl-arachidonoyl-glycerol (18:2 to 20:4) 1 ratio (OR = 0.870, 95%CI = 0.823~0.918, *P*_FDR_= 2.37×10^-5^) was associated with a decreased risk of AS. Heterogeneity was detected in 1-stearoyl-GPE (18:0), 1-palmitoyl-2-arachidonoyl-GPE (16:0/20:4), 1-oleoyl-2-arachidonoyl-GPE (18:1/20:4), and Linoleoyl-arachidonoyl-glycerol (18:2/20:4) 2, while no obvious horizontal pleiotropy was found. We found no evidence linking genetic liability to AS with the identified 61 blood metabolites in the reverse MR analysis (Table S12). The results of “leave-one-out” analysis confirmed the reliability of the MR analysis (Figure S5). The scatter plots illustrated the overall effect of blood metabolites on AS (Figure S6).

### The causal relationship between immune cells and AS

**Figure 5:**
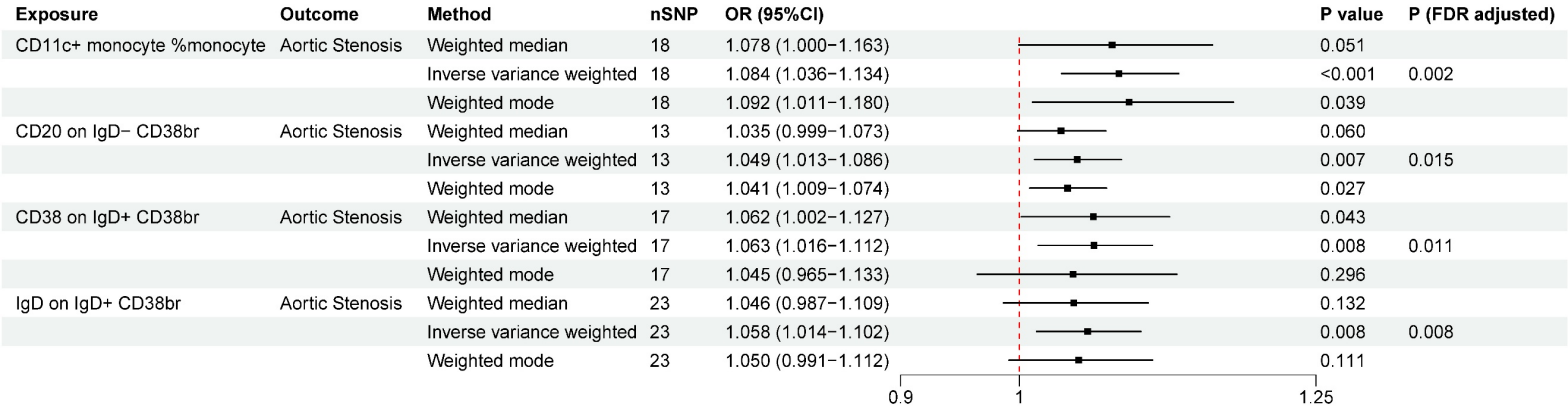
Two-sample mendelian randomization estimations showing the effect of immune cells on aortic stenosis. Only the results of the IVW, weighted median, and weighted mode analysis methods were shown in the figure, and the results of other methods can be found in Table S13. nSNP: total number of instrumental variables used for analysis. *P* (FDR adjusted) < 0.05 was considered statistically significant.

A total of four immunophenotypes were positively correlated with AS both at the nominal significance level of 0.05 and after FDR correction (Figure. 5), including CD11c^+^ monocyte %monocyte (cDC panel, OR = 1.084, 95%CI = 1.036 ~ 1.134 , *P*_FDR_= 0.002), CD20 on IgD^-^ CD38^br^ (B cell panel, OR = 1.049, 95%CI = 1.013 ~ 1.086, *P*_FDR_= 0.015), CD38 on IgD^+^ CD38^br^ (B cell panel, OR = 1.063, 95%CI = 1.016 ~ 1.112, *P*_FDR_= 0.011), IgD on IgD^+^ CD38^br^ (B cell panel, OR = 1.058, 95%CI = 1.014 ~ 1.102, *P*_FDR_= 0.008). Across these outcomes, the B-cell panel emerged as the most significantly associated factor with AS. No pleiotropy was detected by MR-PRESSO for the immune cells analyzed in the primary analysis. In pleiotropy analysis, the *P*-value of CD20 on IgD^-^ CD38br was below 0.05, while *P*-values of the other three immune cells were greater than 0.05. The reverse MR analysis indicated no reverse causal relationship between immune cells and AS (Table S14). The results of “leave-one-out” analysis confirmed the reliability of the MR analysis (Figure S7). The scatter plots demonstrated the overall effect of immune cells on AS (Figure S8).

### The mediation effect of inflammatory proteins, blood metabolites and immune cells in the causal relationship between gut microbiota and AS

It is widely recognized that gut microbiota plays a significant role in the development of various diseases by influencing metabolism and immune function. We performed MR analysis to determine the causal effect of the AS-associated microbial features on the potential mediator (inflammatory proteins, blood metabolites and immune cells) and followed by TSMR.

Two gut microbiota-inflammatory proteins-AS combinations were tested, indicating that higher relative abundance of family *Oscillospiraceae* (OR = 0.841, 95%CI = 0.747 ~ 0.948, *P* = 0.005) and species* Oscillibacter_unclassified* (OR = 0.842, 95%CI = 0.747 ~ 0.949, *P* = 0.005) were associated with decreased blood neurturin concentration (Table S15). However, no significant mediation effect was detected (mediation effect of family *Oscillospiraceae* = 0.0365(-0.00403, 0.077), *P* = 0.078; mediation effect of species* Oscillibacter_unclassified* = 0.0366(-0.00393, 0.0772),* P* = 0.077).

We explored the potential meditating roles of 61 blood metabolites, among which 27 metabolites may increase the risk of AS and the other 34 were negatively correlated with AS. In order to detect more potential meditating roles of blood metabolites, we included a total of 61 metabolites which were shown related to AS at significant level of 0.05. When evaluating the causal effects of GM on blood metabolites, 31 blood metabolites were related to 10 gut microbiota taxa or pathways (Table S16), generating 46 potential gut microbiota-metabolites-AS combinations. In the following mediation analysis, only 10 combinations (involving nine blood metabolites and four gut microbiotas and two pathways) were included based on the standard mentioned above. However, our results indicated that blood metabolites were not mediators in the pathway from gut microbiotas and AS (Table S17).

In MR analysis of gut microbiota and immune cells, all gut microbiota associated with AS were unrelated with immune cells associated with AS (Table S18), which means the effect of gut microbiota on AS may not mediated by immune cells.

## Discussion

**Figure 6:**
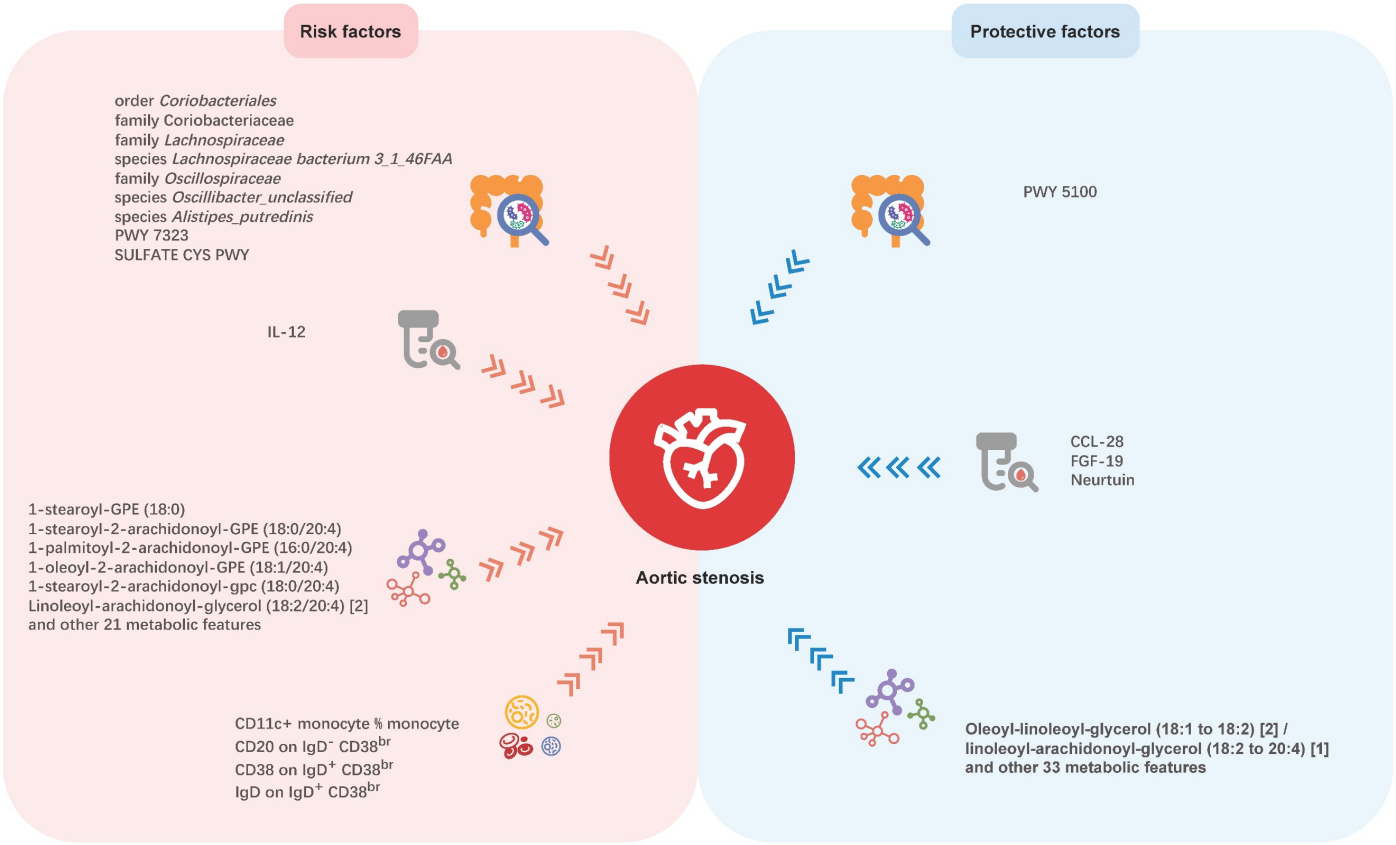
Mendelian randomization analyses show causal effects of gut microbial features, inflammatory proteins, blood metabolites, and immune cell traits on Aortic stenosis. PWY 5100: pyruvate fermentation to acetate and lactate II; PWY 7323: superpathway of GDP-mannose-derived O-antigen building blocks biosynthesis; SULFATE CYS PWY: superpathway of sulfate assimilation and cysteine biosynthesis; CCL-28: C-C motif chemokine ligand 28; FGF-19: fibroblast growth factor 19; IL-12: interleukin-2.

Although previous studies have revealed the association between gut microbiota and AS, the causal relationship remains unclear, due to the intrinsic defects of the observational study. Thus, our study aimed to investigate the causal links between genetically predicted gut microbiota characteristics and AS. In this large-scale and comprehensive MR study, we explored causal relationships between 412 gut microbial features and AS, 91 inflammation-related proteins with AS, 1,400 blood metabolites and AS, and 731 immune cell traits and AS. The potential mediation roles of inflammatory proteins, blood metabolites and immune cells were also been tested in the causal relationship between gut microbiota and AS. We found nine microbial features, four inflammatory proteins, 91 blood metabolites, and four immune cell traits having causal associations with AS, which may offer new insights for future investigations (Figure. 6). But no mediating roles of inflammatory proteins, blood metabolites, and immune cells was detected in the causal association between gut microbiota and AS.

In the present study, most of the AS-related microbiotas are from order* Clostridiales*, class *Clostridia* in phylum *Firmicutes*. Family *Oscillospiraceae* and species* Oscillibacter_unclassified* are members of the family *Oscillospiraceae* in order *Clostridiales* and are positively related to AS. It is noteworthy that the genus *Oscillospira*, an important component of human gut microbiota which has attracted attention from researchers, is also a member of family *Oscillospiraceae,* but is classified into family *Ruminococcaceae*. The genus* Oscillospira* is capable of producing various short-chain fatty acids dominated by butyrate 33,34, and has been proved to have protective effect of many diseases, especially obesity 32,33 and obesity-related chronic inflammatory and metabolic diseases^33^,^37^,^38^. This may be attributed to its negative correlation with chronic inflammation 39. But there are some evidences that it is positively associated to some central nervous system disorders [37,38]and degenerative diseases 33,42-44. Therefore, it is reasonable to assume that different species in family *Oscillospiraceae* may play varying roles in human health. In the original GWAS study for gut microbial traits 23, only two menmbers of family *Oscillospiraceae* were included (genus *Oscillibacter* and species* Oscillibacter_unclassified*), and genus* Oscillospira* was not included. Therefore, the associations between family *Oscillospiraceae,* genus* Oscillospira* and AS still remain to be explored and more research is required to reveal the underlying mechanisms. Although family *Coriobacteriaceae* did not show significant relation with AS, order *Coriobacteriales* seems to be a risk factor for AS, suggesting other family members in order *Coriobacteriales* may be related to AS. Family *Lachnospiraceae* and species *Lachnospiraceae bacterium_3_1_46FAA* may increases the risk of AS. Family* Lachnospiraceae* has been determined to be associated with diabetes, obesity, liver diseases, kidney diseases, as well as inflammatory bowel disease, by altering glucose and lipid metabolism, modulating immune system and inflammation 45-47. Limited study has been carried out on the function of species *Lachnospiraceae bacterium_3_1_46FAA*. Further studies are needed to reveal if and how it is implicated in AS development. Taken together, these microbiotas have not been reported yet and may serve as potential targets for the treatment of AS. *Prevotella copri*, a member of *Bacteroidetes,* seemed to play roles in immunity and inflammation, and has been correlated with atherosclerosis, hypertension, and heart failure. Liu et al. 19 performed 16S rRNA sequencing on fecal samples and indicated that gut microbiota in patients with cardiac valve calcification, which was characterized by increased *Prevotella copri*, was different from both healthy controls and patients with coronary artery disease. Interestingly, our study did not identify a significant causal correlation between* Prevotella copri* and AS. This discrepancy may be partly attributed to the small sample size and study design employed by Liu et al 19, which included only 19 patients with cardiac valve calcification and did not distinguish aortic valve calcification from the calcification of other cardiac valves. Besides, cross-sectional studies can only reveal associations and are not capable of establishing causality. Therefore, longitudinal studies with a lager sample size are needed to validate these associations. Additionally, it should be noted that species *Alistipes_putredinis* (a member of genus *Alistipes*, family *Rikenellaceae*, order *Bacteroidales*, class *Bacteroidetes*, phylum *Bacterodetes*) was determined to be a risk factor of AS. The genus *Alistipes* has drawn increasing interests of researchers and been linked with cardiovascular diseases such as atrial fibrillation, congestive heart failure, and atherosclerosis cardiovascular disease 48. Although there are accumulating evidences, the involvement of *Alistipes* in cardiovascular diseases is still contradictory 49, and our study may bring some new insights. Altogether, our study highlights the potential of microbiome-targeting therapy for AS prevention and treatment. But when we interpret the influence of gut microbiota on AS, it should be noted that the gut microbiota itself may be influenced by a variety of factors, such as dietary habits, medication use, and comorbid conditions, and it is also in a state of dynamic change. Besides, other traditional factors which has been widely accepted as risk factors of AS, including older age, male sex, hypertension, smoking, diabetes, and elevated serum lipoprotein(a) levels, still need to be prioritized when we discuss the etiology of AS.

Aortic valve interstitial cells (VICs), playing a crucial role in the development of AS, are a heterogeneous population of cells including fibroblasts and smooth muscle cells 12,50. Cytokines have been demonstrated to influence the differentiation of VICs, therefore we explored the link between inflammatory proteins and AS. As a result, CCL-28, FGF-19 and neurturin were detected to be protective factors, while IL-2 was linked to increased risk of AS. In previous studies, CCL-28, FGF-19 and IL-2 were mostly investigated in immune disorders and tumor treatment 51-54, and neurturin was mainly discussed in muscular, neurodegenerative and psychiatric disorders 55,56. Here we revealed their potential effects on AS for the first time.

Concerning the relationship between blood metabolites and AS, 1-stearoyl-2-arachidonoyl-gpc (18:0/20:4) and 1-stearoyl-2-arachidonoyl-GPE (18:0/20:4) are both glycerophospholipids generated by the amalgamation of stearic acid and arachidonic acid (AA), constituting different phosphoryl groups. These glycerophospholipids have been found to be related with ischemic heart diseases and left ventricular diastolic dysfunction, and inversely associated with *Intestinimonas_massilliensis* 57. However, clinical study revealing the relationship between specific gut microbiota and the glycerophospholipids and glycerolipids associated with AS are relatively lacking, and more evidence comes from MR study. An MR study 58 suggested that genus *Eubacterium nodatum* was negatively associated with 1-stearoyl-2-arachidonoyl-GPE (18:0/20:4), 1-palmitoyl-2-arachidonoyl-GPE (16:0/20:4), and 1-oleoyl-2-arachidonoyl-GPE (18:1/20:4). Genus *Holdemanella* and genus *Peptococcus* were also showed to be related with the level of 1-palmitoyl-2-arachidonoyl-GPE (16:0/20:4) 59. Regrettably, we could not verify these relationships because the primary GWAS study 23 we used did not include the microbiota mentioned above. The oleoyl-linoleoyl-glycerol (18:1 to 18:2) 2 to linoleoyl-arachidonoyl-glycerol (18:2 to 20:4) 1 ratio was associated with a decreased risk of AS, but the oleoyl-linoleoyl-glycerol (18:1 to 18:2) 2 level was not associated with AS (*P*_FDR_ > 0.05). To be noted, the genetic features of linoleoyl-arachidonoyl-glycerol (18:2 to 20:4) 1 level were not included in the original GWAS study. Therefore, it seems that linoleoyl-arachidonoyl-glycerol (18:2 to 20:4) 1 level may be a risk factor of AS. All the 7 metabolites which showed most significant causal effects on AS are composed of AA. Studies have unveiled that AA could be metabolized by three distinct enzyme systems: cyclooxygenases (COXs, also known as PGG/H synthases), lipoxygenases, and cytochrome P450 enzymes (ω-hydroxylases and epoxygenases), producing a wide range of biologically active fatty acid mediators 60, which also have been verified to be important in the development and progression of cardiovascular diseases 61. This investigation postulates that these glycerolipids may contribute to the development of AS. Regretfully, there is currently short of clinical research concerning these metabolites, highlighting the need for further exploration in the future. Trimethylamine N-oxide (TMAO), a well-studied gut microbiota metabolite, has been found to accelerate the progression of AS and was associated with adverse outcome after transcatheter aortic valve implantation 18,62. Some studies also highlighted the roles of tryptophan derivatives, especially indoxyl sulfate and serotonin, in osteogenic differentiation and inflammation of AS^16^. We also included TMAO, some tryptophan derivatives, and some bile acid derivatives in our analysis. However, none of these metabolites was found to be associated with AS at the genetic level. As we know, these metabolites are highly influenced by dietary components and dependent on hepatic metabolism. Therefore, the increase of these metabolites in patients with AS may not be determined by genetics, but rather influenced by diet or other metabolic factors.

Our study found that the risk of AS increased with an elevation in the proportion of CD11c^+^ monocyte. CD11c^+^ monocyte mostly consists of intermediate monocytes and nonclassical monocytes 63, comprising only 10% of total blood monocytes, yet secreting high levels of proinflammatory cytokines 64-66. They have also been found to be related with high body mass index (BMI), hyperlipidemia and cardiovascular diseases 65,67-70, including atherosclerosis and coronary artery diseases. Our study adds new evidence of the possibly crucial role of these monocytes in the development of AS. Within B cell panel, our analysis detected a significant relationship between CD38 and IgD on IgD^+^ CD38^br^ B-cells and AS, as well as a causal relation between CD20 on IgD^-^ CD38^br^ B-cells and AS. CD38 is expressed on peripheral B cells during early differentiation and activation, and has been verified to be linked with multiple B-cell-related diseases 71. IgD is expressed on the surface of mature B cells after they have encountered their specific antigen for the first time, and orchestrates a surveillance system at the interface of immunity and inflammation 72,73. CD20 is regarded as an effective therapeutic target for a majority of B-cell malignancies, but the precise physiological role and function in disease progress remain unclear 74. However, it should be noted that only immune cells in peripheral blood were included in the original GWAS study. Concerning the participation of immune cells in aortic valve diseases, the aortic valve is inhabited by tissue-resident macrophages, mast cells, dendritic cells and T cells 75-77, and T cells were recognized as important inflammatory drivers of progressive calcific aortic valve disease 78,79. Monocytes can also be recruited after aortic valve endothelial injury 80. But the role of B cells in the development of AS has received less attention.

Our study had some limitations. Firstly, this study only included European populations. Variations in genetic factors, environmental exposures, and lifestyles across different populations could impact the relationship between gut microbiota, metabolites, and AS, limiting the generalizability of the findings to other ethnic or regional groups. Secondly, the analysis did not completely account for potential confounders, such as comorbidities and antibiotics, which could change the intestinal microbiota composition and alter metabolite profiles, influencing the link between gut microbiota and AS. Additionally, the Mendelian randomization approach itself has limitations. It can only analyze association between exposures and outcomes at genetic level, but genetic influences may be compensated for by other biological processes over time, potentially weakening the observed genetic effects. Overreliance on MR may reinforce genetic determinism, overshadowing the importance of environmental and lifestyle factors in disease etiology. Combining MR with other methodologies, such as randomized controlled trials and mechanistic studies, can provide a more comprehensive understanding of causal relationships and inform clinical and public health interventions. Future studies are warranted to address these limitations, explore mechanisms behind gut microbiota-AS relationship and offer new perspectives on AS prevention and treatment.

## Conclusion

Using Mendelian randomization analysis, we identified several gut microbiota, inflammatory proteins, blood metabolites, and immune cell traits genetically associated with AS. However, no significant mediating effects have been identified for the gut bacteria-mediated inflammatory proteins, blood metabolites or immune cell traits associated with AS. These findings provide new insights into the pathophysiology of AS and establish a theoretical basis for future therapeutic approaches.

## Supplementary Material

Supplementary figures.

Supplementary tables.

## Abbreviations

AS: aortic stenosis
MR: Mendelian randomization
TSMR: two-step Mendelian randomization
IV: instrumental variables
GWAS: genome-wide association study
IVW: inverse-variance weighted
OR: odds ratio
CI: confidence interval
FDR: false discovery rate
MR-PRESSO: MR Pleiotropy RESidual Sum and Outlier
CCL-28: C-C motif chemokine ligand 28
FGF-19: fibroblast growth factor 19
IL-2: interleukin-2
VIC: valve interstitial cells
TMAO: Trimethylamine N-oxide
SNP: single nucleotide polymorphism
