# Supplementary material for: Exploring the Relationship Between Gut Microbiota and Aortic Stenosis: Role of Inflammatory Proteins, Blood Metabolites, and Immune Cells
Source: Int J Med Sci. 2025 Mar 10;22(8):1750–61. doi: 10.7150/ijms.110392 (PMC11983298; doi:10.7150/ijms.110392)
Supplement: Supplementary file 1 — Supplementary figures. [file ijmsv22p1750s1.pdf]

Descriptions of Supplementary Figures and Tables

Figure S1 The leave-one-out analysis for gut microbiota on aortic stenosis.

Figure S2 The scatter plots show overall effect of blood metabolites on aortic stenosis.

Figure S3 The leave-one-out analysis for inflammatory proteins on aortic stenosis.

Figure S4 The scatter plots show overall effect of gut microbiota on aortic stenosis.

Figure S5 The leave-one-out analysis for blood metabolites on aortic stenosis.

Figure S6 The scatter plots show overall effect of inflammatory proteins on aortic stenosis.

Figure S7 The leave-one-out analysis for immune cells on aortic stenosis.

Figure S8 The scatter plots show overall effect of immune cells on aortic stenosis.

Table S1. Detailed information for genome-wide association studies involved in the present Mendelian randomization study.

Table S2. The instruments of gut microbiota.

Table S3. The instruments of aortic stenosis.

Table S4. The instruments of inflammatory proteins.

Table S5. The instruments of blood metabolites.

Table S6. The instruments of immune cells.

Table S7. Mendelian randomization analysis on the causal effect of gut microbiota on AS.

Table S8. Mendelian randomization analysis on the causal effect of AS on gut microbiota.

Table S9. Mendelian randomization analysis on the causal effect of inflammatory proteins on AS.

Table S10. Mendelian randomization analysis on the causal effect of AS on inflammatory proteins.

Table S11. Mendelian randomization analysis on the causal effect of blood metabolites on AS.

Table S12. Mendelian randomization analysis on the causal effect of AS on blood metabolites.

Table S13. Mendelian randomization analysis on the causal effect of immune cells on AS.

Table S14. Mendelian randomization analysis on the causal effect of AS on immune cells.

Table S15. Mendelian randomization analysis on the causal effect of AS-associated gut microbiota on AS-associated inflammatory proteins.

Table S16. Mendelian randomization analysis on the causal effect of AS-associated gut microbiota on AS-associated blood metabolites.

Table S17. The mediation analysis of blood metabolites in the causal relationship between gut microbiota and AS.

Table S18. Mendelian randomization analysis on the causal effect of AS-associated gut microbiota on AS-associated immune cells.

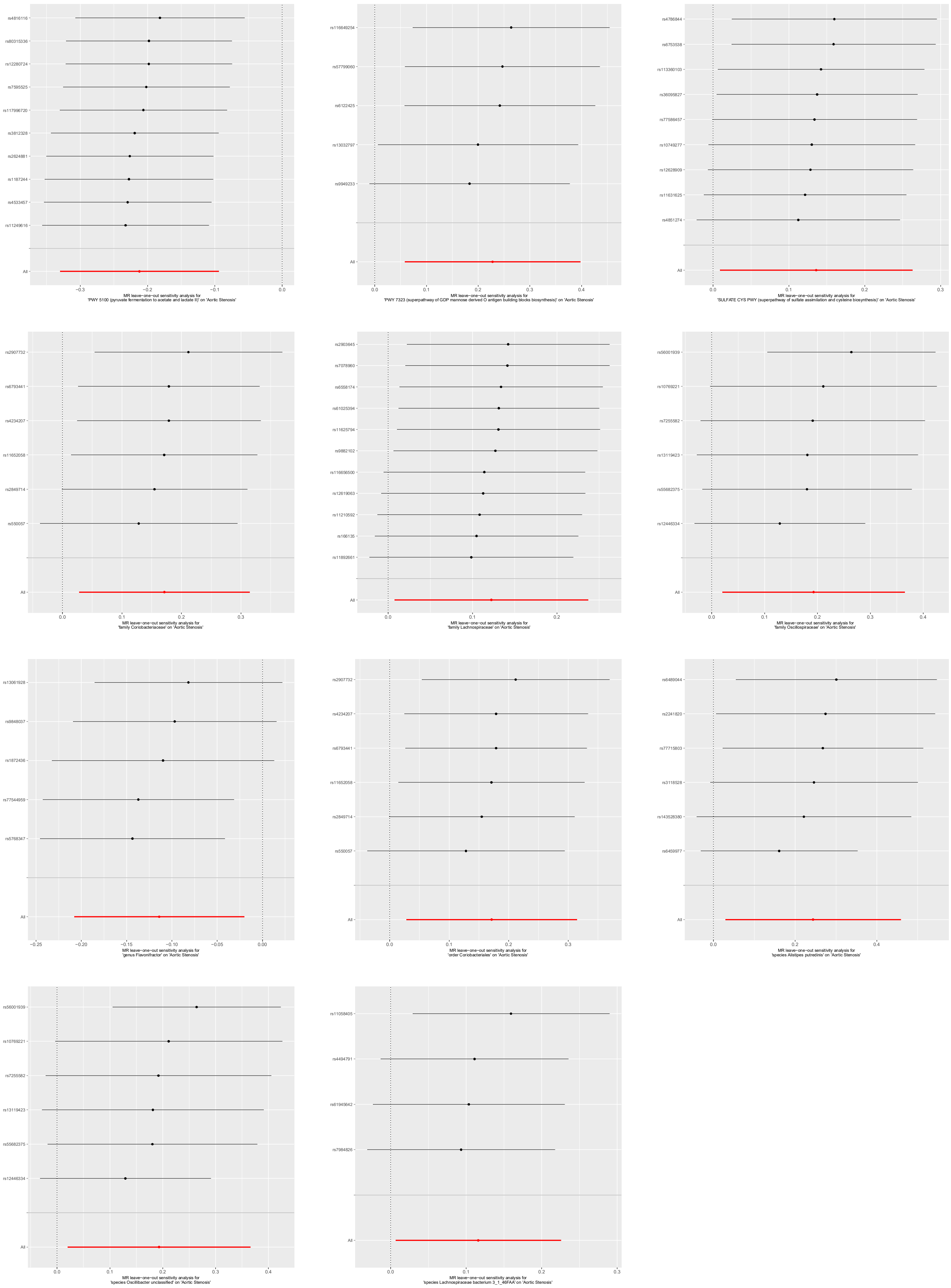

Figure S1. The leave-one-out analysis for gut microbiota on aortic stenosis.

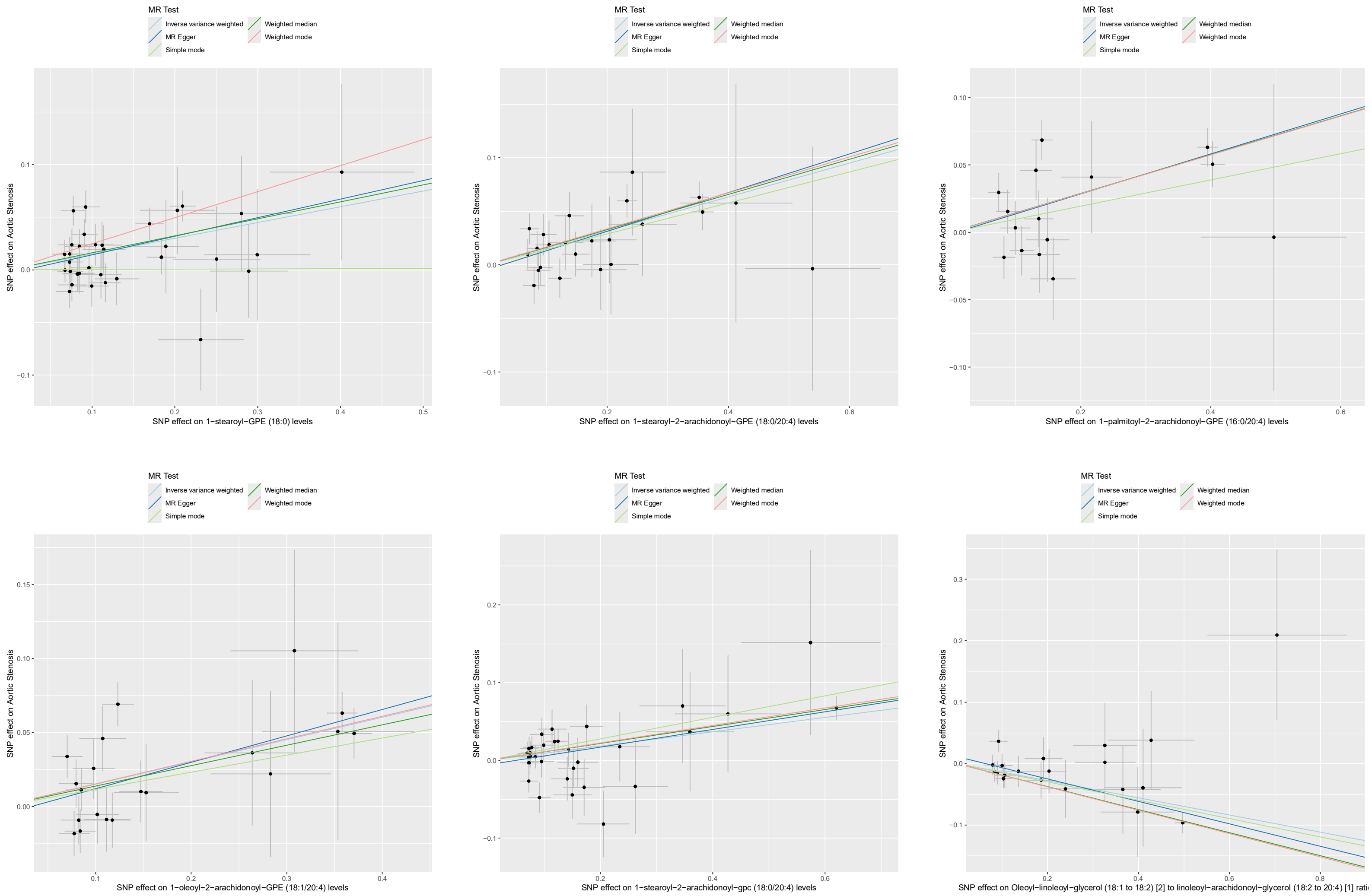

Figure S2. The scatter plots show overall effect of blood metabolites on aortic stenosis.

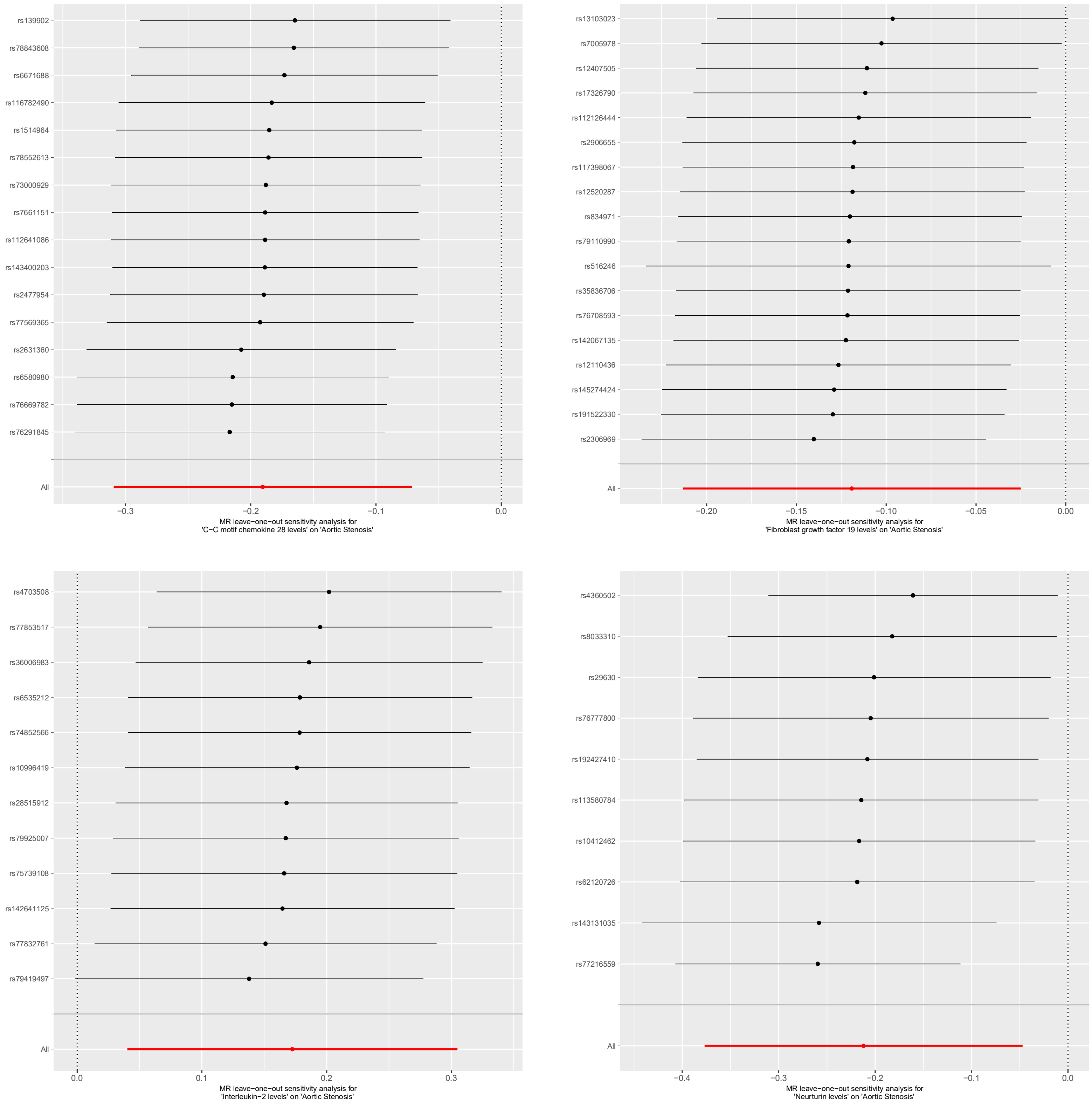

Figure S3. The leave-one-out analysis for inflammatory proteins on aortic stenosis.

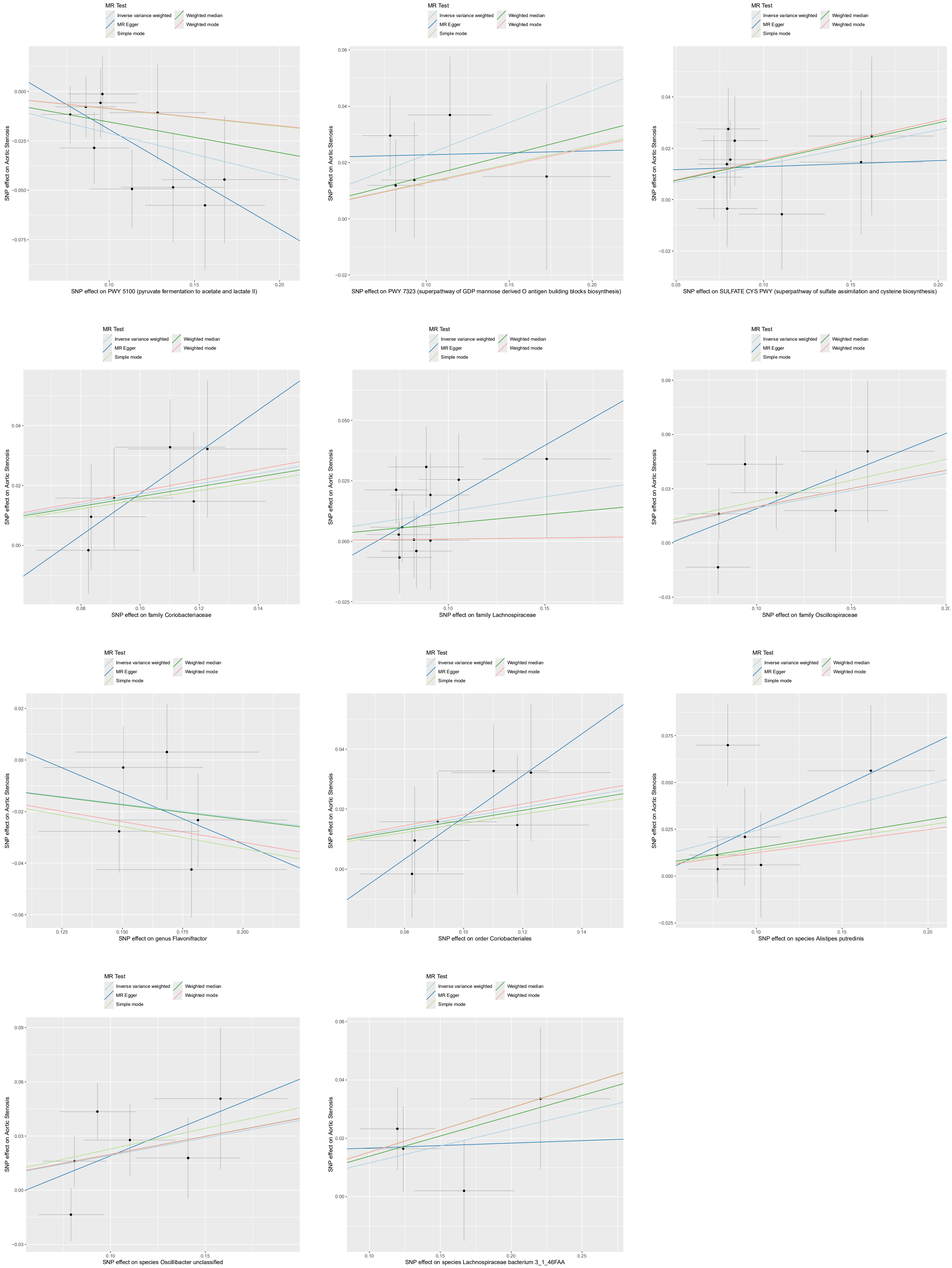

Figure S4. The scatter plots show overall effect of gut microbiota on aortic stenosis.

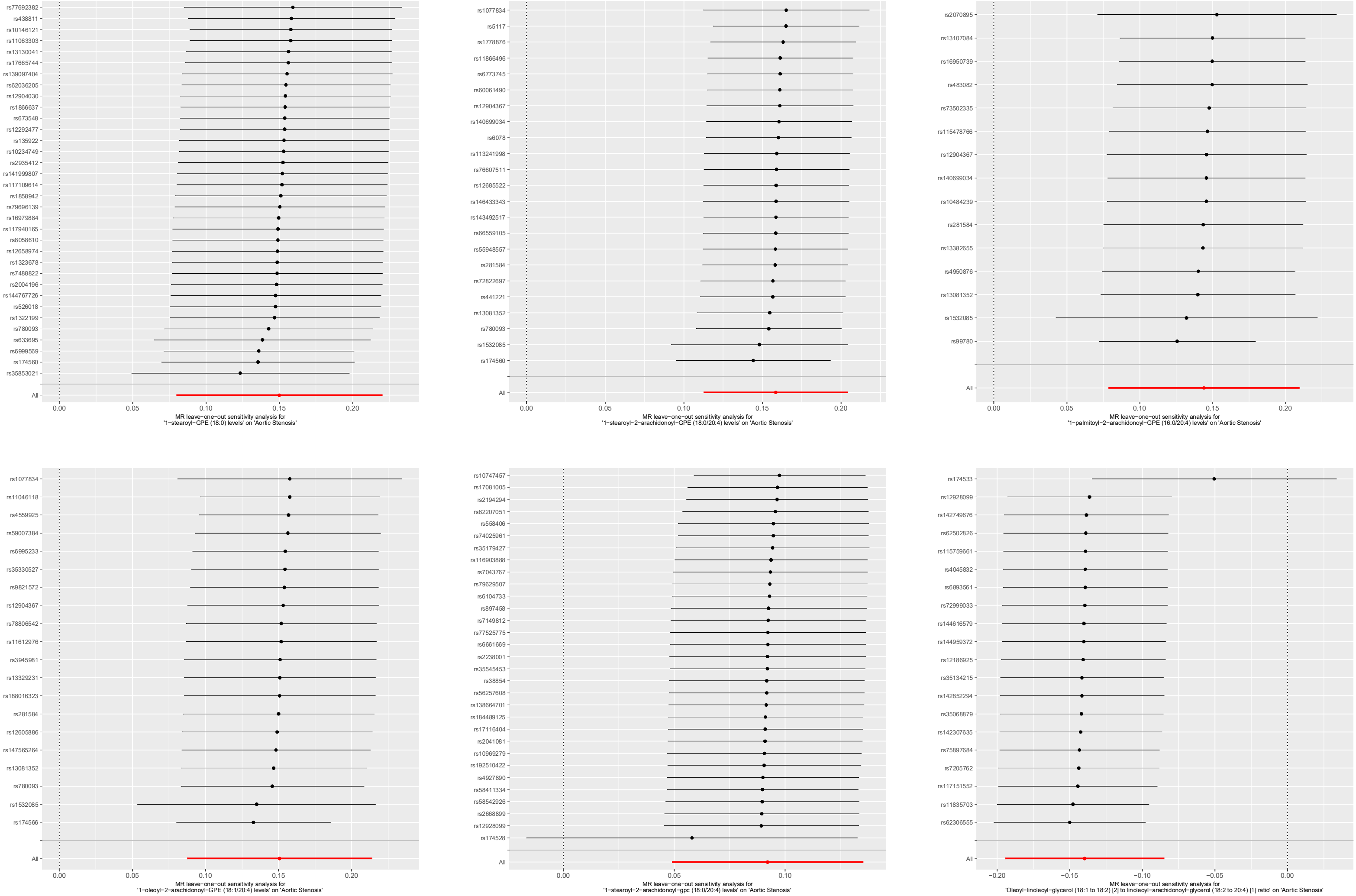

Figure S5. The leave-one-out analysis for blood metabolites on aortic stenosis.

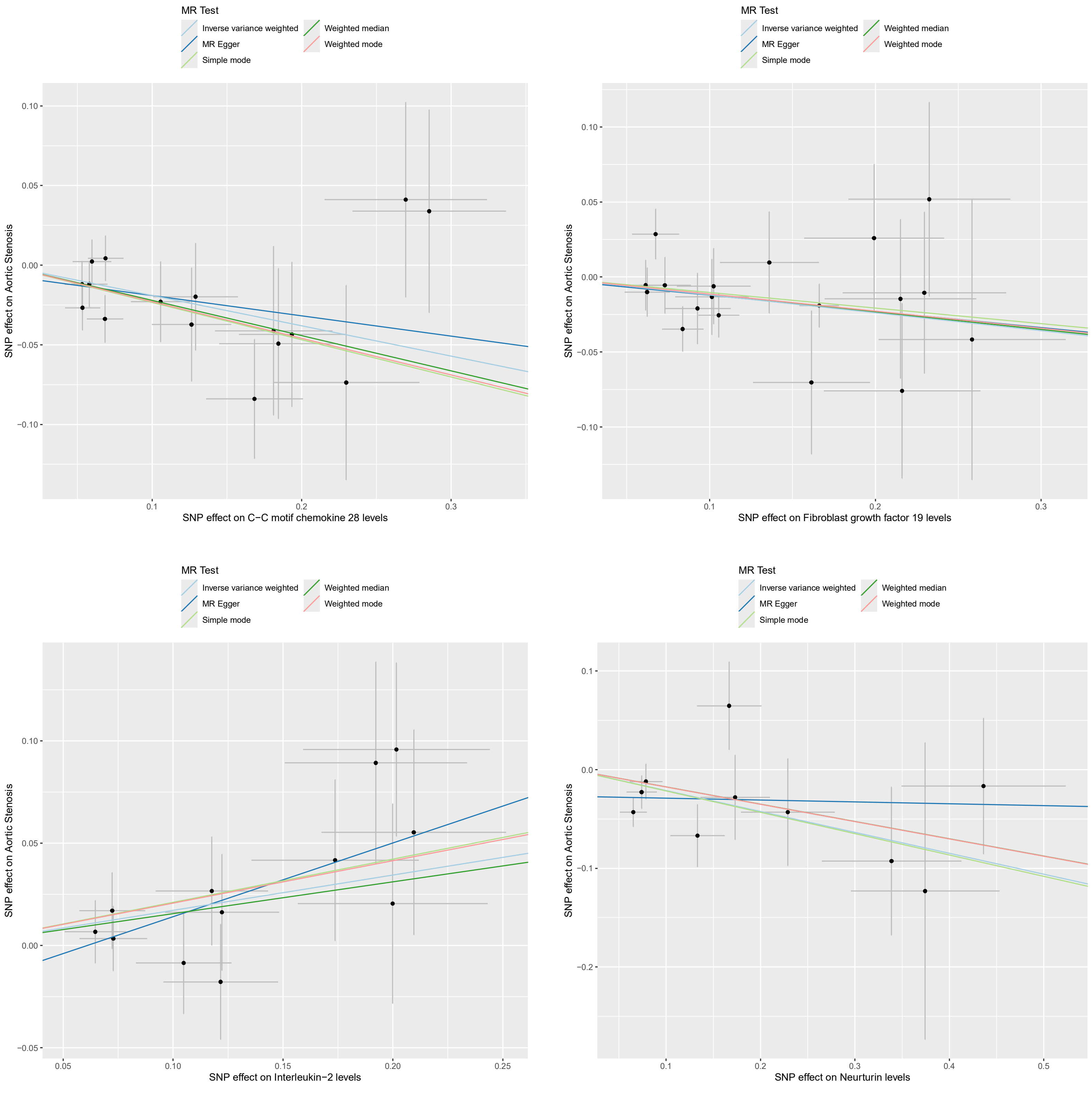

Figure S6. The scatter plots show overall effect of inflammatory proteins on aortic stenosis.

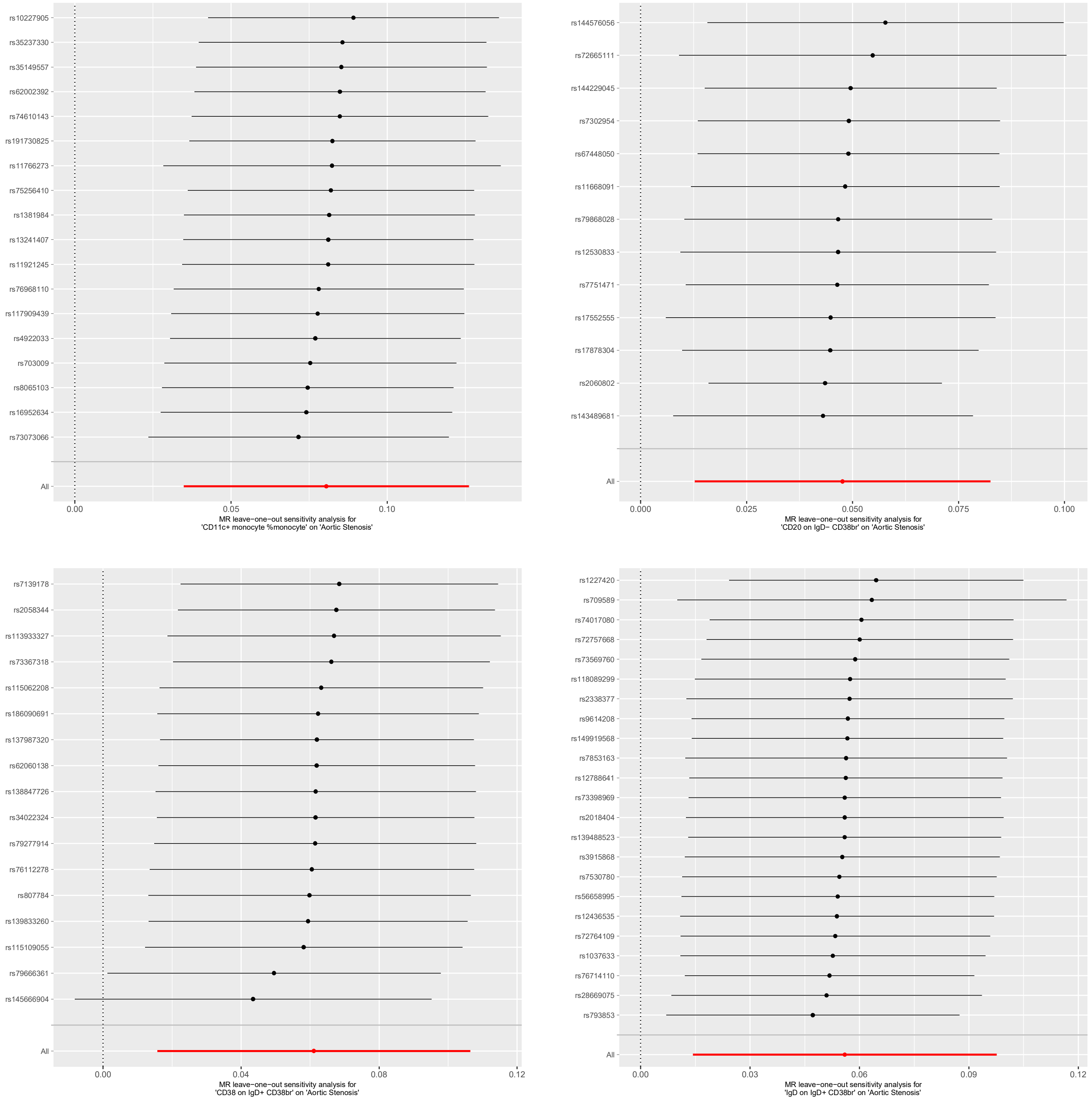

Figure S7. The leave-one-out analysis for immune cells on aortic stenosis.

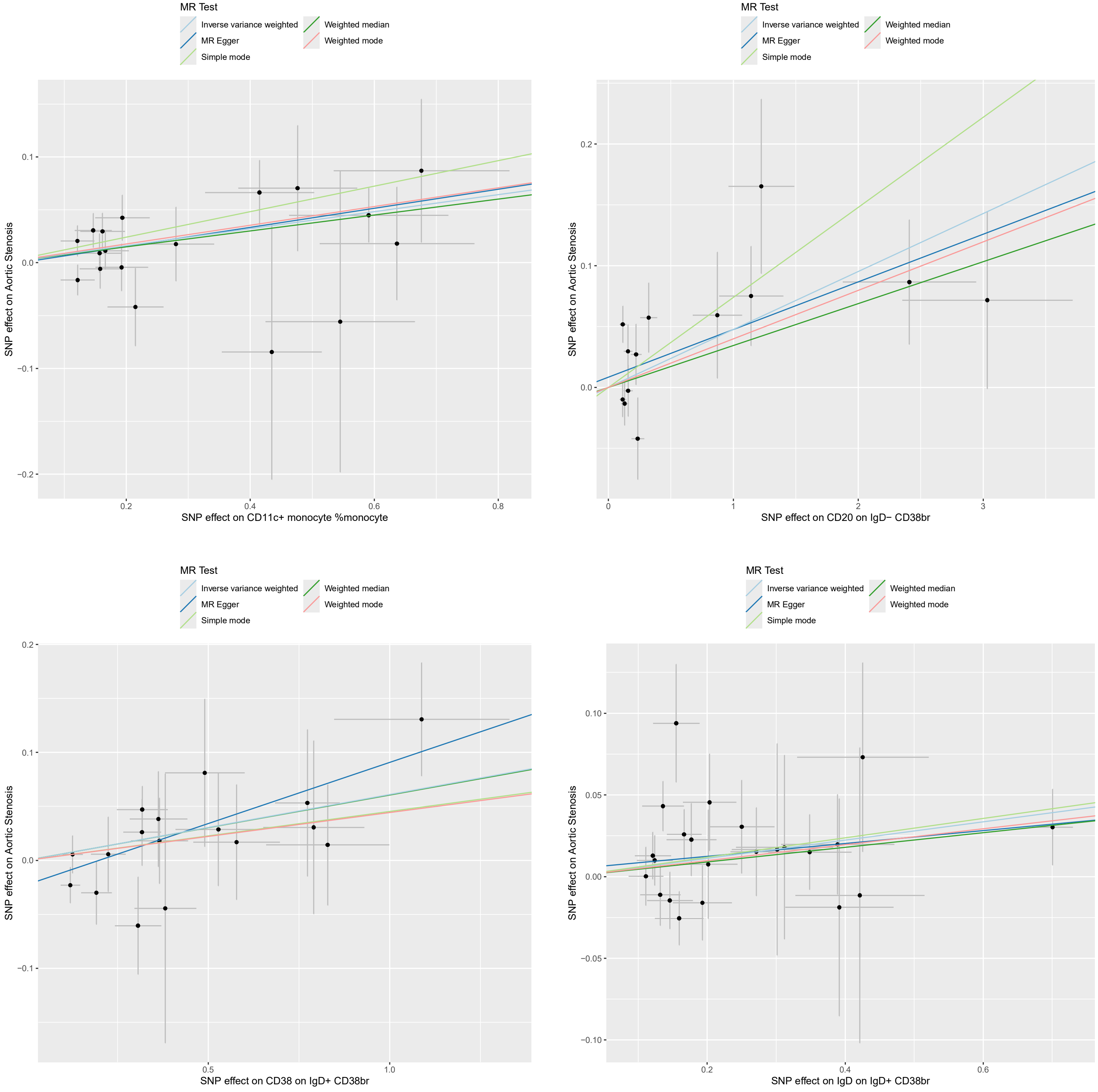

Figure S8. The scatter plots show overall effect of immune cells on aortic stenosis.
